# Doxycycline and Monocaprin In Situ Hydrogel: Effect on Stability, Mucoadhesion and Texture Analysis and In Vitro Release

**DOI:** 10.3390/gels5040047

**Published:** 2019-12-09

**Authors:** Venu Gopal Reddy Patlolla, William Peter Holbrook, Sveinbjorn Gizurarson, Thordis Kristmundsdottir

**Affiliations:** 1 Faculty of Pharmaceutical Sciences, University of Iceland, Hofsvallagata 53, 107 Reykjavik, Iceland; vgr1@hi.is (V.G.R.P.); sveinbj@hi.is (S.G.); 2 Faculty of Odontology, University of Iceland, Vatnsmýrarveg 16, 101 Reykjavík, Iceland; phol@hi.is

**Keywords:** doxycycline, monocaprin, stability, oral mucosa, drug delivery, MMP

## Abstract

The aim of this study was to develop a stable aqueous formulation containing a combination of doxycycline and monocaprin in clinically relevant concentrations. Increase in expression of Matrix metalloproteinases (MMPs) and microbial role in oral diseases is well established and the combination of above active ingredients could be potentially beneficial in treatment of oral mucosal conditions. The hydrogels containing different concentrations of doxycycline and monocaprin in the presence and absence of stabilizing excipients were developed and their stabilities were studied at 4 °C for up to 1 year. The drug–drug interaction was evaluated using Fourier-transform infrared spectroscopy (FTIR). The addition of monocaprin on doxycycline in situ hydrogel’s mucoadhesiveness, texture properties and drug release mechanism was studied. The addition of monocaprin negatively affected the doxycycline stability and was concentration dependent, whereas monocaprin was stable up to 1 year. Doxycycline did not interfere with the anti-Candidal activity of monocaprin. Furthermore, the presence of monocaprin significantly affected the formulation hardness, compressibility and adhesiveness. Monocaprin and doxycycline release followed zero order kinetics and the release mechanism was, by anomalous (non-Fickian) diffusion. The addition of monocaprin increased the drug release time and altered the release mechanism. It is possible to stabilize doxycycline in the presence of monocaprin up to 1 year at 4 °C.

## 1. Introduction

The oral mucosa is easily accessible and convenient site for local drug delivery. The bioavailability of active ingredients as well as their therapeutic outcome, increases when administered locally rather than via the systemic route. The oral mucosa is up to 4000 times more permeable relative to the skin [1,2] and has excellent vasculature for drug diffusion into the systemic circulation through capillaries and venous drainage and thereby prevent the drugs from the harsh gastrointestinal environment as well as avoiding the first-pass metabolism. This route may also reduce the systemic side effects. Virtual lack of Langerhans cells makes the oral mucosa tolerant to potential allergens and less susceptible to toxicity from locally applied agents [3].

Hydrogels are composed of cross-linked polymeric networks with high water content (70–99%). They offer excellent controlled release properties which can be tailored by spatial and temporal control and also mimics the soft tissue, improving the biocompatibility [4,5,6]. Hydrogels are also utilized to protect the labile drugs from degradation and to encapsulate the drug molecules by different physicochemical interactions [6]. Hydrogels enhance the bioadhesion to the mucosal lining, which plays a key role in drug delivery through nasal and oral route [7]. Poloxamers are triblock copolymer, non-ionic surfactants composed of two hydrophilic polyoxypropylene (POP) units and a core hydrophobic polyoxyethyle (PEO) unit. Poloxamer hydrogels shows reversible thermal gelation properties due to their ability to self-assemble into micelles in aqueous solutions [8].

Some oral mucosal conditions require frequent clinical interventions, which can lead to treatment resistance over time. Safe alternative treatments that are optimized for oral mucosa are required. In a previous study by Skulason et al., it was showed that cold sores were effectively treated using combination of doxycycline and monocaprin, where doxycycline acts as matrix metalloproteinase (MMP) inhibitor and monocaprin as an anti-viral agent [9]. The healing times and pain reduction was significantly improved, compared to the existing treatments. In another clinical trial by Skulason et al., a hydrogel containing doxycycline was very effective in treating aphthous ulcer and the healing time were significantly faster than existing treatments [10]. However, doxycycline stability was a drawback in a long-term clinical study. Patlolla et al. successfully stabilized doxycycline in aqueous formulation for long-term shelf storage [11] but the effect of presence of monocaprin on doxycycline stability is unknown.

Monocaprin is a lipid that has a broad microbicidal activity against various bacteria [12,13,14] (e.g., gram positive bacteria (*Group A streptococcus, Staphylococcus aureus* and *Group B streptococcus*) and gram negative bacteria (*Neisseria gonorrhoeae* and *Chlamydia trachomatis* and *Helicobacter pylori* [14])), enveloped viruses [12,15,16] (e.g., herpes simplex virus type 1 (HSV-1), herpes simplex virus type 2 (HSV-2), human immunodeficiency virus type 1 (HIV-1) and vesicular stomatitis virus) and yeast [17,18] (e.g., *Candida albicans*), that can be clinically useful in treating oral infections. Infections usually accompany inflammation and doxycycline helps to downgrade inflammation by inhibiting MMPs. Doxycycline is clinically approved for MMP inhibition by the US Food and Drug Administration (FDA) [19] and its inhibitory activity on MMPs is nonspecific [20,21].

The aim of this study was to incorporate monocaprin to doxycycline hydrogels and to study the effect of monocaprin on doxycycline stability as well as to stabilize active ingredients as doxycycline is susceptible to oxidation and epimerization [11] whereas, monocaprin is susceptible to hydrolysis and cleavage of fatty acid i.e., capric acid from glycerol and also acyl-migration in solutions [22]. Also, to study the drug interactions and to evaluate the effect of addition of monocaprin on formulation mucoadhesiveness, texture properties, drug release and mechanism of drug release.

## 2. Results

### 2.1. Drug Interaction

#### 2.1.1. FTIR

**Figure 1:**
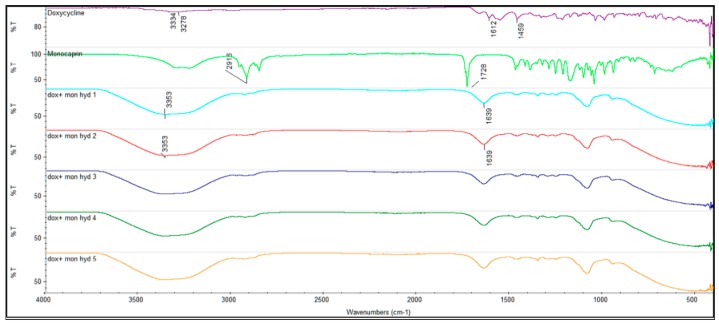
FTIR spectra of doxycycline, monocaprin and hydrogels 1–5 (dox + mon hyd1 = hydrogel1, dox + mon hyd2 = hydrogel2, dox + mon hyd3 = hydrogel3, dox + mon hyd4 = hydrogel4 and dox + mon hyd5 = hydrogel5) containing doxycycline and monocaprin.

The overlay of the FTIR transmission spectra of doxycycline and monocaprin and hydrogels 1–5 is shown in Figure 1. The FTIR spectra of all the hydrogels (hyd1-5) showed similar characteristic O-H stretch 3353 cm^−1^ and band, 1639 cm^−1^, irrespective of the presence of doxycycline, monocaprin and stabilizing excipients. Doxycycline hyclate sample showed characteristic –OH stretch at 3334 cm^−1^, 3278 cm^−1^ (-NH), 1612 cm^−1^ (–C=O) and 1459 cm^−1^ (–CH_2_) bending. Monocaprin showed bands at 2916 cm^−1^ and 1728 cm^−1^. 

#### 2.1.2. Anti-Microbial Studies

**Figure 2:**
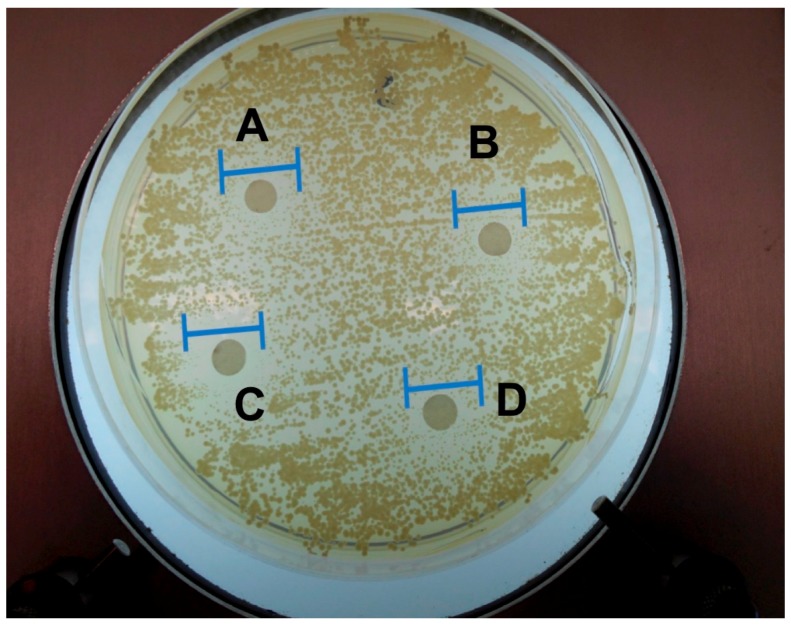
In vitro anti-Candida test for monocaprin hydrogels “A” (only monocaprin 0.5%), Hydrogel “B” (only monocaprin 1%), Hydrogel “C” (doxycycline 0.15% + monocaprin 0.5%) and Hydrogel “D” (doxycycline 0.15% + monocaprin 1%), on *Candida albicans.*

The zone of inhibition (ZOI) of all the tested hydrogels were similar, hydrogel A 9.3 ± 1.24 mm, hydrogel B 9.7 ± 0.94 mm, hydrogel C 10 ± 0 mm and hydrogel D 9.7 ± 0.47 mm (Figure 2). The ZOI values for 0.5% and 1% monocaprin formulations and also in presence of doxycycline were almost similar, indicating that doxycycline did not interfere with the antimicrobial activity of monocaprin and monocaprin at 0.5% *w*/*w* was equally efficient as 1% *w*/*w*. 

### 2.2. Monocaprin UHPLC-CAD Method Validation

The method showed good linearity with correlation coefficient *R*^2^ = 0.9995. The precision values for intraday AUC, %RSD ranged from 0.54% to 0.86% and inter-day AUC, %RSD ranged between 0.60% and 0.85%. The LOD and LOQ were found to be 0.000045% *w*/*v* and 0.00014% *w*/*v* respectively. The percentage recovery for 0.1% *w*/*v* was 99.5 ± 0.31, for 0.15% *w*/*v* was 100.8 ± 0.77 and for 0.2% *w*/*v* was 101.8% ± 0.50. 

### 2.3. Stability Studies

#### 2.3.1. Doxycycline Stability (Effect of Increase in Concentration on Stability)

**Figure 3:**
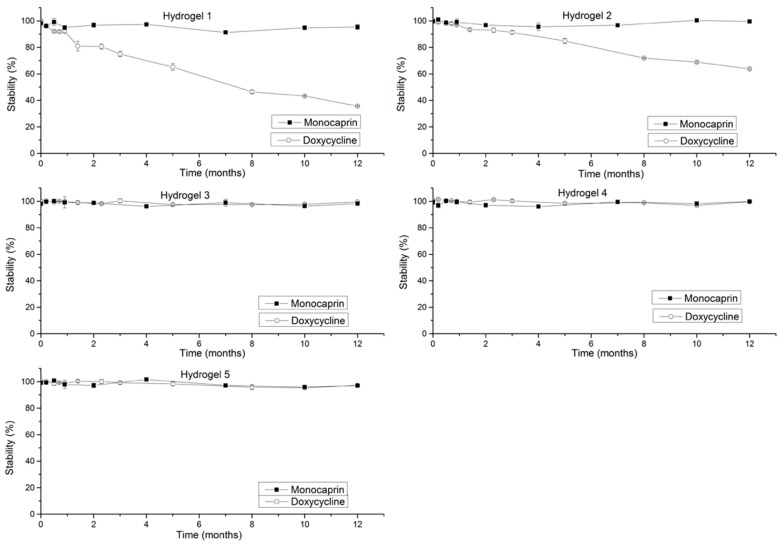
Stabilities of doxycycline and monocaprin in hydrogels 1–5 (hydrogel 1 (0.1% doxycycline and 0.5% monocaprin), hydrogel 2, 3, 4 and 5 (0.15% doxycycline) and 1%, 0.5%, 1% and 0.5% of monocaprin respectively, at 4 °C.

The stability of doxycycline was negatively affected in the presence of monocaprin and with further increase in concentration of monocaprin (Figure 3, Hydrogel 2 vs. Hydrogel 1) the doxycycline stability was improved. Degradation product, 4-epidoxycycline was observed due to epimerization of doxycycline. 

Monocaprin is a lipophilic compound which forms colloid in aqueous solutions and when added to poloxamer solution it occupies the lipophilic core of the surfactant micelle and doxycycline also might occupy the lipophilic core, which was evident from the stabilized doxycycline formulations by Patlolla et al. [11] but addition of monocaprin did not seem to affect the doxycycline distribution coefficient and is also evident as no oxidation degradation product was seen and further increase in monocaprin concentration showed positive effect on doxycycline stability.

#### 2.3.2. Monocaprin Stability (Effect of Increase in Concentration on Stability)

Monocaprin was found to be stable in the presence of doxycycline irrespective of concentrations (doxycycline 0.1% and 0.15% and monocaprin 0.5% and 1%), by end of 1 year at 4 °C (Figure 3).

*Effect of stabilizing excipients:* Monocaprin stability was unaffected by presence of stabilizing excipients (Figure 3, Hydrogels 3 and 4). From Figure 3, hydrogels 3 and 4, doxycycline was stabilized over a period of 1 year at 4 °C by addition of antioxidants, chelating agent and a complexing agent MgCl_2_ in poloxamer solution at pH 6.55 (Patlolla et al.) [11].

*Effect of preservatives:* Presence of preservatives did not affect the stabilities of doxycycline and monocaprin (Figure 3, Hydrogel 5).

### 2.4. Mucoadhesion

**Figure 4:**
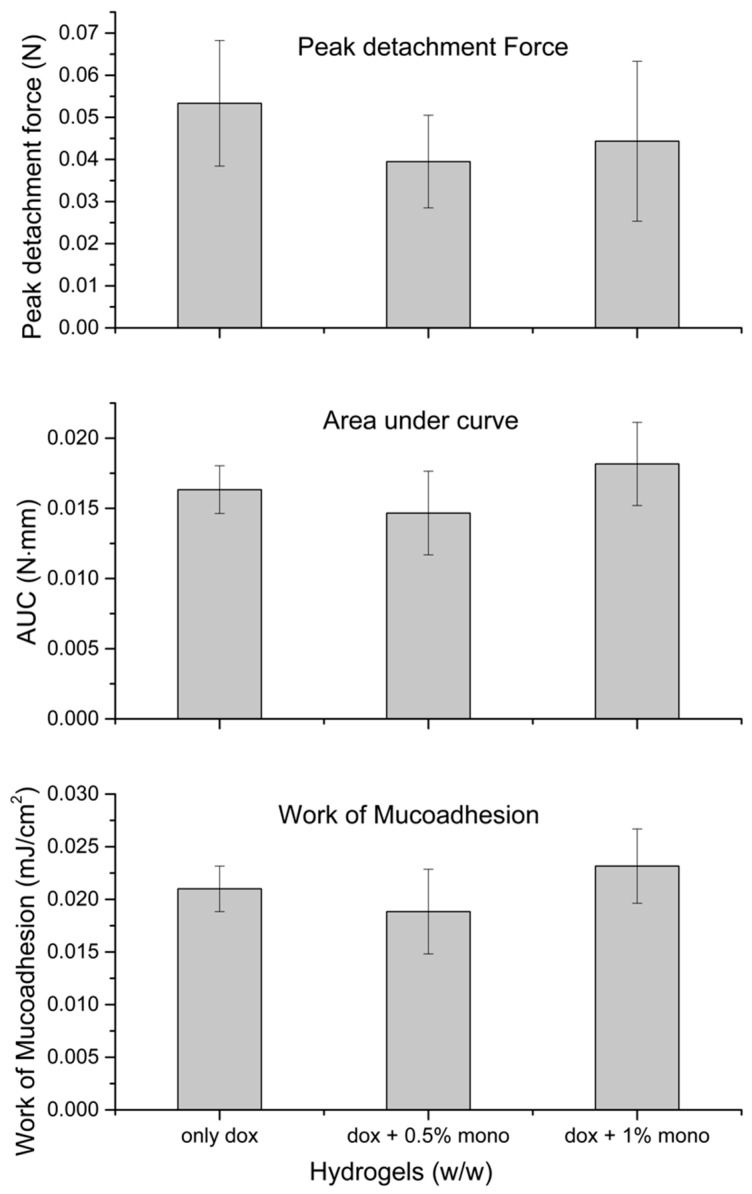
In vitro mucoadhesion test for hydrogel: Comparison of work of mucoadhesion, area under curve (AUC) and peak detachment force for hydrogels containing, only doxycycline 0.1% (only dox) [23], for hydrogel 1 containing doxycycline 0.1% and monocaprin 0.5% (dox + 0.5% mono) and for hydrogel 2 containing doxycycline 0.15% and monocaprin 1% (dox + 1% mono).

From Figure 4, addition of monocaprin appears to affect the mucoadhesion capacity of the hydrogel and the effect was also concentration dependent. At 0.5% monocaprin, the peak detachment force and work of mucoadhesion values decreased, indicating reduction in formulation retention time, but not significantly (*p* = 0.15 for work of mucoadhesion and *p* = 0.37 for peak detachment force). However, at 1% the work of mucoadhesion increased and the peak detachment force decreased (not significantly). The 1% monocaprin formulation showed improved AUC values but not significant (*p* = 0.14). 

### 2.5. Texture Analysis

**Figure 5:**
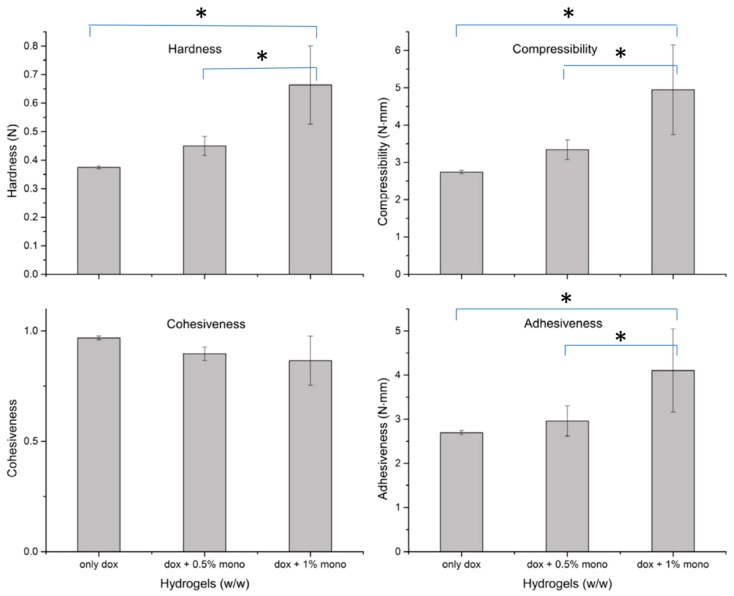
In vitro texture profile analysis: Hardness, compressibility, cohesiveness, adhesiveness for hydrogels containing doxycycline 0.1% (only dox) [23], hydrogel 1 with doxycycline 0.1% and monocaprin 0.5% (dox + 0.5%mono) and hydrogel 2 containing doxycycline 0.15% and monocaprin 1% (dox + 1% mono). * *p* < 0.05, One way ANOVA Tukey HSD *post hoc* test.

The texture properties of hydrogels containing only doxycycline and in presence of increasing concentrations of monocaprin is shown in Figure 5. The hardness, compressibility and adhesiveness of hydrogel (Hydrogel 2) containing 1% monocaprin was significantly (*p* < 0.05, one way ANOVA, Tukey HSD) higher than hydrogel containing only doxycycline [23] and hydrogel with doxycycline and monocaprin 0.5% (Hydrogel 1). Presence of monocaprin irrespective of concentration, did not significantly affect (*p* = 0.07) the cohesiveness of the formulation.

### 2.6. In Vitro Release (Doxycycline and Monocaprin) Release Mechanism

**Figure 6:**
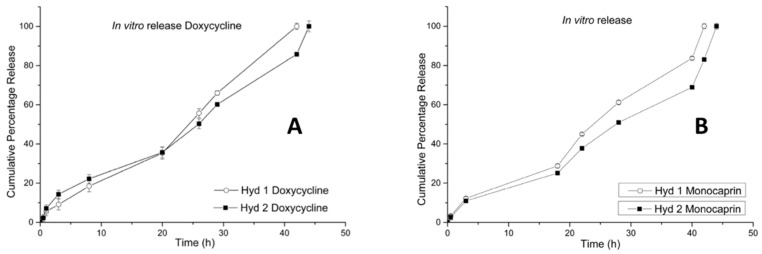
In vitro release study of doxycycline (**A**) monocaprin (**B**) from hydrogel 1 containing doxycycline 0.1% and monocaprin 0.5% and hydrogel 2 containing doxycycline 0.15% and monocaprin 1%.

The monocaprin and doxycycline release from the poloxamer-HPMC-povidone matrices was found to follow zero order kinetics. The presence of monocaprin doubled the drug release time for both doxycycline as well as monocaprin, compared to hydrogel containing only doxycycline [23] and it was also concentration dependent. With the increase in concentration of monocaprin the drug release (both doxycycline and monocaprin) was extended by 2 hours (Figure 6). The kinetic model showed a good correlation for both zero order kinetics and the Korsmeyer-Peppas model and the release exponents, *n* = 0.67 and *n* = 0.68 for monocaprin and *n* = 0.74 and *n* = 0.71 for doxycycline in hydrogel 1 and 2 respectively, indicated that the drug release mechanism is by anomalous (non-Fickian) diffusion [24].

## 3. Discussion

Doxycycline alone was highly stable in the aqueous hydrogel formulation (Patlolla et al.) [11], but when monocaprin was added, its stability was only 30% by end of 1 year at 4 °C. Monocaprin, however, was highly stable and was not affected by doxycycline. In the hydrogels, an epimerization occurred of doxycycline since the only degradation product seen was 4-epidoxycycline. Doxycycline was stabilized by addition of excipients [11,25,26,27,28], Mgcl_2_ where Mg^2+^ chelates with the epimerization prone site 4-N(CH_3_)_2_ of doxycycline and hinders the steric rearrangement, also suitable antioxidants, sodium metabisulfite and sodium thiosulfate were added. Additional chelating agent disodium edetate was added. It was possible to stabilize doxycycline even in the presence of monocaprin and stability was unaffected by further increase in concentration of monocaprin. Although monocaprin is known to have antimicrobial effects, the addition of additional preservatives did not affect monocaprin and doxycycline stabilities. Though FTIR study of the hydrogels was not conclusive to rule out drug–drug interaction, the in vitro anti-candida test showed that presence of doxycycline did not impact the microbicidal activity of monocaprin. Furthermore, the use of surfactants could hinder the antimicrobial activity by entrapment of the monocaprin inside the surfactant micelles [22], but interestingly this effect was not observed for poloxamer’s. 

The novelty of the study was using charged aerosol detector for monocaprin stability studies. UHPLC-CAD quantification of monocaprin was accurate compared to the UV (HPLC-UV) detectors especially due to, monocaprin has low UV absorption maxima value of 208 nm [22] which is a troublesome UV detection region and interference from excipients and solvents and base line noise could interfere with the quantification leading to high standard deviation values. The CAD method for monocaprin showed very good peak resolution and stable base line compared to the UV method.

The main idea behind the combination of monocaprin and doxycycline was to benefit from the MMP inhibitory effects of doxycycline as well as the antimicrobial effects of monocaprin. Monocaprin and the low concentration of doxycycline does not alter the normal microbial flora [29,30,31,32], but affects many pathogenic microbes such as candida. Therefore, this may be used to prevent the development of candidiasis. When monocaprin is included in denture formulations it has been found to effectively inhibited candida in the mouth of geriatric patients (Holbrook et al.) [33] and it has also been shown to function as denture disinfectant (Thorgeirsdottir et al.) [18] though the inhibitory effect was short lived.

The addition of monocaprin decreased the gelation temperature to around 27 °C, instead of 33–34 °C [23] and the hydrogels were still usable when stored in refrigerated conditions and directly applied to the site. Also adjusting the gelation temperature was found to alter the HLB value of the poloxamer surfactant which might affect stability of doxycycline. The gelation temperature was affected by the addition of monocaprin but the effect was not concentration dependent and stabilizing excipients did not affect the gelation temperature. Monocaprin significantly increased the formulation hardness, compressibility indicating decrease in retention but also increased the adhesiveness. Presence of monocaprin also doubled the drug release time and effected the release mechanism.

## 4. Conclusions

Hydrogels containing combination of doxycycline and monocaprin were developed and stabilized for long-term storage, while preserving their pharmacological activities. The physical characteristics of these formulations may allow them to be used as a carrier for therapeutics to treat many oral diseases. Here, we evaluated the robustness and the physical and pharmaceutical characteristics of hydrogel formulations containing the MMP inhibition compound, doxycycline as well as the potential microbicide monocaprin, for the treatment of oral infections. 

## 5. Materials and Methods

### 5.1. Materials

Doxycycline hyclate and monocaprin analytical standards, sodium thiosulfate, sodium metabisulfite, methyl parahydroxybenzoate, propyl parahydroxybenzoate, acetonitrile, *tert*-butanol and povidone were obtained from Sigma-Aldrich (Darmstadt, Germany). Difco Sabouraud dextran agar was obtained from Becton Dickinson & Co (Franklin Lakes, NJ, USA). Poloxamer 407 and poloxamer 188 were obtained from BASF (Ludwigshafen, Rhineland-Palatinate, Germany). Doxycycline hyclate was provided from Hovione (Taipa, Macau). Monocaprin was obtained from Danisco (Copenhagen, Denmark). Hydroxypropyl methylcellulose (HPMC) (Methocel™ K4M, Premium) was obtained from Colorcon (Kent, England). Disodium edetate (EDTA) was obtained from Riedel-de Haën (Seelze, Germany). Magnesium chloride hexahydrate was obtained from Merck (Darmstadt, Germany). 2-Hydroxypropyl-ß-cyclodextrins (HPßCD) was a generously provided from Roquette Pharmaceuticals (Lestrem, France). Potassium dihydrogen phosphate (KH_2_PO_4_) and tetrabutyl ammonium-bisulfite were obtained from Fluka (Darmstadt, Germany).

### 5.2. Manufacture of Hydrogels

The hydrogels were manufactured by using the cold method [34]. Poloxamers were dissolved in the refrigerated Milli-Q water followed by the mucoadhesive polymers, HPMC and povidone. The excipients, antioxidants, chelating agent and MgCl_2_. Doxycycline and monocaprin were added and the pH of the hydrogels was manually adjusted using 1M HCl and 1M NaOH solutions. The final weight was made-up by addition of Milli-Q water. The hydrogels were manufactured in the following order, Hydrogels 1–5, consisted of following doxycycline concentration: 0.1%; 0.15%; 0.15%; 0.15%; and 0.15% *w*/*w* respectively and following monocaprin concentrations: 0.5%; 1%; 0.5%; 1%; and 0.5% *w*/*w* respectively. Hydrogel 1 and 2 consisted of Poloxamer 407 (21% *w*/*w*), 188 (10 % *w*/*w*), HPMC (0.25% *w*/*w*) and Povidone (0.25% *w*/*w*). Hydrogel 3 and 4 consisted of additional 2 antioxidants (sodium metabisulfite (0.32% w/w), sodium thiosulfate (0.32% *w*/*w*), chelating agent, EDTA (0.2% *w*/*w*) and divalent magnesium ions (MgCl_2_) (1:4 molar ratio to doxycycline) Hydrogel 5 consisted of additional preservative agents, methyl parahydroxybenzoate (0.2% *w*/*w*) and propyl parahydroxybenzoate (0.2% *w*/*w*). In addition, hydrogels A and B containing 0.5% *w*/*w* and 1% *w*/*w* monocaprin respectively were manufactured for the *in vitro* anti-Candidal study. Hydrogels A and B consisted of Poloxamer 407 (21% *w*/*w*), poloxamer 188 (10% *w*/*w*), HPMC (0.25% *w*/*w*) and povidone (0.25% *w*/*w*).

### 5.3. Gelation Temperature 

The gelation temperature was adjusted by method described by Patlolla et al. [11]. Hydrogel was placed in a test tube and immersed in water bath attached to a thermostat at 20 °C. The gelation was examined at every 1 °C increments by tilting the test tubes and gelation was considered when meniscus no longer moved. 

### 5.4. Fourier-Transform Infrared Spectroscopy (FTIR)

The FTIR spectra of the hydrogels was studied by Nicolet iZ10 MX from Thermo Scientific using Omnic software from Thermo fisher scientific (Waltham, MA, USA). The spectra of hydrogels were studies between wavenumbers, 4000 and 400 cm^−1^.

### 5.5. Stability Studies

The stability studies for doxycycline and monocaprin were studied at established time until 1 year. The hydrogels stability was studied at 4 °C. The effect of monocaprin on doxycycline stability and vice versa was evaluated.

### 5.6. Analysis of Doxycycline

Doxycycline was quantified using an HPLC method described in Ph. Eur. (Ph. Eur. 8th Edition, 2014 (8.0)). A reversed-phase HPLC system obtained from Dionex Softron GmbH (Germering, Germany) equipped with p680 pump with DG-1210 degasser, an ASI-100 autosampler and VWD-3400 UV-Vis detector. The column was Agilent PLRP-S styrene-divinyl benzene copolymer 250 mm × 4.60 mm (8 µm). The column was maintained at 60 °C, and the flow rate was 1 mL/min. Standards and sample of 20 µL were withdrawn for injection after diluting with 0.01 M hydrochloric acid solution. All the measurements were conducted in triplicate and the standard curve was obtained from 5 serial dilutions. 

### 5.7. Analysis of Monocaprin 

Monocaprin quantification was carried out by reverse-phase ultra-high-performance liquid chromatography system (UHPLC) from Dionex Softron GmbH (Germering, Germany) and Corona^®^ ultra RS charged aerosol detector (CAD). The Ultimate 3000 series, was equipped with a Pump (LPG-3400SD) and a built-in degasser, WPS-3000 auto-sampler, TCC-3100 column compartment and a Corona^®^ ultra RS detector with nebulizer temperature control set at 25 °C. The stationary phase was Cosmosil C18 (4.6 mm × 150 mm) equipped with a Security Guard pre-column, Phenomenex C18 (10 mm × 4.6 mm) [35]. The mobile phase composed of acetonitrile and water (58:42) [22]. The retention time was about 5.5 min with flow rate at 1 mL/min. The temperature of column compartment was set at 25 °C while the sample (vials) compartment was set at 7 °C. The injection volume of 20 µL was withdrawn from the samples and standards after suitable dilution with the mobile phase. Chromatograms were evaluated using ChromeleonR version 7.2 SR4 (ThermoFisher Scientific). The validation of the UHPLC-CAD, was performed according to ICH guidelines by following Q2(R1) [36] and tested for,

*Linearity:* A calibration curve was made with concentration ranging from 3 µg/mL to 60 µg/mL. All the samples were injected 3 times.
*Precision:* The intraday and inter-day precision was measured for 3 different concentrations of monocaprin ranging from 5 µg/mL to 25 µg/mL and each injected for 5 times. The area under curve (AUC) was obtained for all the injections and precision is expressed as relative standard deviation (%RSD).
*Accuracy:* Accuracy was measured as the percentage recovery. Three different concentrations of monocaprin (0.1, 0.15 and 0.2% *w*/*v*) in 5 % *w*/*v* HPBCD solutions, were injected (*n* = 3) after suitable dilution and the average percentage recovery was calculated.
*Limit of detection (LOD) and limit of quantification (LOQ):* The LOD and LOQ were calculated from linearity obtained from 5 dilutions, 1.1, 2.2, 3.2, 4.3 and 5.4 µg/mL solutions, by using following Equations (1) and (2) [36],

(1) $$LOD=\frac{\mathrm{standard}\;\mathrm{deviation}\;\mathrm{of}\;\mathrm{response}}{slope}\;\;X\;3.3$$

(2) $$LOQ=\frac{\mathrm{standard}\;\mathrm{deviation}\;\mathrm{of}\;\mathrm{response}\;}{slope}\;X\;10$$

### 5.8. In Vitro Mucoadhesion

The hydrogels mucoadhesive properties were studied using Texture Analyzer TA-XT2i (Stable Microsystems, UK) equipped with a 5 Kg load cell as described previously [23]. A sample hydrogel (3 gm) beaker (5 mL) was attached to the texture analyzer platform using a double adhesive tape after the gelation was induced by placing in an oven at 37 °C before each test, covered with a parafilm to prevent evaporation. DuoDerm® artificial membrane coated with a layer of mucus (17% crude mucin, pH adjusted to 6.0 and viscosity 0.039 Pa·s [37]) was used to simulate the mucus membranes, adhered to the moving probe (cylindrical graphite probe (P/10)). During test, the probe was lowered to just touch in the surface (trigger force = 0.003N) of hydrogel and contact force was set at 0.005 N and probe speed, 0.1 mm/sec. Once the artificial membrane contacts the hydrogel surface, a constant contact force was applied for 90 sec, allowing the mucoadhesive bonds between the hydrogel and membrane. After the test, the probe was pulled away from the hydrogel surface with a probe speed, 0.1 mm/sec until 10 mm apart (height). The force required to separate the membrane from hydrogel surface was recorded as a function of elongation and both maximum strength and area under force-time curve were obtained. Six repeated measurements were taken for each hydrogel.

(3) $$\mathrm{Work}\;\mathrm{of}\;\mathrm{mucoadhesion}\;\frac{AUC}{\pi r^{2}}=\left(\frac{mJ}{cm^{2}}\right)$$

The tensile strength was measured as the peak force obtained during the detachment of test hydrogel from the membrane.

Work of mucoadhesion was measured (Equation (3) [38]) as the work done to separate the hydrogel from the mucus membrane.

### 5.9. Texture Profile Analysis (TPA)

The hydrogel texture properties were studied as described by Patlolla et al. [23], using Texture analyzer TA-XT2i equipped with, 5 Kg load cell. The TPA properties were studied after inducing gelation at 37 °C in an oven. The hydrogel sample was adhered on the Texture analyzer platform using a double adhesive tape and probe used was, cylindrical graphite probe (P/10), with 10 mm diameter. The trigger force for the probe was 0.010 N, the test speed was 0.5 mm/s and pretest and posttest speeds, 0.1 mm/s. The time interval of 5 sec was set between the 2 compression cycles (operating in TPA2 mode) and the probe travel depth was 10 mm for 2 cycles. Six repeated measurements were noted for each hydrogel sample.

*Hardness:* was obtained as the maximum force required to achieve a deformation or defined as maximum peak force in the first cycle of compression [23,39,40,41].
*Cohesiveness:* was measured as internal structural strength, which sustains strong interconnections with certain level of resistance to rupture. Cohesiveness was calculated from the ratio of area under curve (AUC) under force-time curve for second compression cycle to the first compression cycle (A2/A1) [23,39,42,43].
*Compressibility:* was measured as work done to achieve deformation of the hydrogel during the first cycle of compression [23,39].
*Adhesiveness:* was measured as, work required to overcome the adhesive forces between the entire surface of probe and hydrogel surface which comes in contact with the probe, while the probe is in retraction mode after the compressibility cycle or also can be defined as negative region of force-time AUC after the first cycle of compression [23,39,42,43].

### 5.10. In Vitro Antimicrobial Study

The antimicrobial activity of in situ hydrogels containing monocaprin alone and in combination with doxycycline were evaluated for *Candida albicans* using sabouraud agar. The *C. albicans* strain was sourced from Faculty of Odontology (University of Iceland, Reykjavik, Iceland). The swab of the cultured *C. albicans* was spread on agar plates and dried. The in situ hydrogels were introduced onto a paper disc and incubated with the agar. The anti-Candidal activity was measured as diameter in mm of the inhibition zone (triplicate measurements).

### 5.11. In Vitro Release

Monocaprin and doxycycline release from the poloxamer hydrogel was studied by membrane-less model [44]. Two grams of the hydrogel was introduced into a test tube without inducing bubbles and allowed the undergo gelation in oven pre-set at 37 °C. The release medium used for monocaprin was simulated saliva solution containing 0.05M KH_2_PO_4_ + 1.25% HPβCD and adjusted to pH 6.75, which was equilibrated to 37 °C [35] whereas for doxycycline the release medium used was simulated saliva solution containing 0.05M KH_2_PO_4_, pH adjusted to 6.75 equilibrated to 37 °C. The test-tubes were secured inside a temperature controlled (37 °C) shaker, set at 100 rpm (Lab-line Orbit Environ-Shaker from Lab-line instruments. Inc., Melrose Park, IL, USA). 10 mL of the release medium was introduced on the solidified hydrogel without causing erosion and aliquots of entire volume was used for sampling at regular intervals. The sampling medium was completely replaced at each sampling intervals. For monocaprin, the samples were further diluted with 58:42 (acetonitrile/water) and analyzed using UHPLC-CAD and for doxycycline the samples were diluted using 0.01 M hydrochloric acid solution. The drug release kinetics and mechanism of drug release was studied from, Equation (4) [45] and Equation (5) [24].

*Zero order*

(4) $$M_{t}=M_{0}+k_{0}t$$

*M_t_* is the amount of drug released at time *t*; *K_0_* is the zero-order release constant and *M_0_* is the amount of drug released into the solution at time *t* = 0, usually *M_0_*= 0.

*Korsmeyer-Peppas (power law model)*

(5) $$\frac{M_{t}}{M_{\infty }}=Kt^{n}$$

where *M_t_/M_∞_* is the fraction of drug released at time “*t*”. *K* is the release rate constant incorporating structural and geometrical characteristics of the device and n is the release exponent characteristic of release mechanism. The release exponent “*n*” value was calculated from the initial 60% of the drug release curve, which was plotted as log cumulative percentage drug release vs. log time [24,46].

### 5.12. Statistics 

RStudio (Version 1.1.463 RStudio, Inc. Boston, MA, USA) was used for the calculation of statistical significance using one-way analysis of variance (ANOVA), Tukey HSD *post hoc* test and *p* < 0.05 was considered statistically significant.

## References

[1] Galey W.R., Lonsdale H.K., Nacht S. (1976). The in vitro permeability of skin and buccal mucosa to selected drugs and tritiated water. J. Invest. Dermatol..

[2] Bala R., Pawar P., Khanna S., Arora S. (2013). Orally dissolving strips: A new approach to oral drug delivery system. Int. J. Pharm. Investig..

[3] Boddé H.E., De Vries M.E., Junginger H.E. (1990). Mucoadhesive polymers for the buccal delivery of peptides, structure-adhesiveness relationships. J. Control. Release.

[4] Arakaki K., Kitamura N., Fujiki H., Kurokawa T., Iwamoto M., Ueno M., Kanaya F., Osada Y., Gong J.P., Yasuda K. (2010). Artificial cartilage made from a novel double-network hydrogel: In vivo effects on the normal cartilage and ex vivo evaluation of the friction property. J. Biomed. Mater. Res. A.

[5] Li J., Illeperuma W.R.K., Suo Z., Vlassak J.J. (2014). Hybrid Hydrogels with Extremely High Stiffness and Toughness. ACS Macro Lett..

[6] Li J., Mooney D.J. (2016). Designing hydrogels for controlled drug delivery. Nat. Rev. Mater..

[7] Peppas N.A., Sahlin J.J. (1996). Hydrogels as mucoadhesive and bioadhesive materials: A review. Biomaterials.

[8] Alexandridis P., Alan Hatton T. (1995). Poly(ethylene oxide)-poly(propylene oxide)-poly(ethylene oxide) block copolymer surfactants in aqueous solutions and at interfaces: Thermodynamics, structure, dynamics, and modeling. Colloids Surf. A Physicochem. Eng. Asp..

[9] Skulason S., Holbrook W.P., Thormar H., Gunnarsson G.B., Kristmundsdottir T. (2012). A study of the clinical activity of a gel combining monocaprin and doxycycline: A novel treatment for herpes labialis. J. Oral Pathol. Med..

[10] Skulason S., Holbrook W.P., Kristmundsdottir T. (2009). Clinical assessment of the effect of a matrix metalloproteinase inhibitor on aphthous ulcers. Acta Odontol. Scand..

[11] Patlolla V.G.R., Holbrook W.P., Gizurarson S., Kristmundsdottir T. (2019). Long-term stabilization of aqueous doxycycline formulations, in mucoadhesive hydrogels for treatment of oral mucosal conditions. Curr. Drug Discov. Technol..

[12] Thormar H., Isaacs C.E., Brown H.R., Barshatzky M.R., Pessolano T. (1987). Inactivation of enveloped viruses and killing of cells by fatty acids and monoglycerides. Antimicrob. Agents Chemother..

[13] Bergsson G., Arnfinnsson J., Steingrimsson O., Thormar H. (2001). Killing of Gram-positive cocci by fatty acids and monoglycerides. APMIS.

[14] Bergsson G., Steingrimsson O., Thormar H. (2002). Bactericidal effects of fatty acids and monoglycerides on Helicobacter pylori. Int. J. Antimicrob. Agents.

[15] Thormar H., Bergsson G., Gunnarsson E., Georgsson G., Witvrouw M., Steingrimsson O., De Clercq E., Kristmundsdottir T. (1999). Hydrogels containing monocaprin have potent microbicidal activities against sexually transmitted viruses and bacteria in vitro. Sex. Transm. Infect..

[16] Kristmundsdottir T., Arnadottir S.G., Bergsson G., Thormar H. (1999). Development and evaluation of microbicidal hydrogels containing monoglyceride as the active ingredient. J. Pharm. Sci..

[17] Bergsson G., Arnfinnsson J., Steingrimsson O., Thormar H. (2001). In vitro killing of Candida albicans by fatty acids and monoglycerides. Antimicrob. Agents Chemother..

[18] Thorgeirsdottir T.O., Kristmundsdottir T., Thormar H., Axelsdottir I., Holbrook W.P. (2006). Antimicrobial activity of monocaprin: A monoglyceride with potential use as a denture disinfectant. Acta Odontol. Scand..

[19] Benjamin M.M., Khalil R.A. (2012). Matrix metalloproteinase inhibitors as investigative tools in the pathogenesis and management of vascular disease. Exp. Suppl..

[20] Liu J., Xiong W., Baca-Regen L., Nagase H., Baxter B.T. (2003). Mechanism of inhibition of matrix metalloproteinase-2 expression by doxycycline in human aortic smooth muscle cells. J. Vasc. Surg..

[21] Goktolga U., Cavkaytar S., Altinbas S.K., Tapisiz O.L., Tapisiz A., Erdem O. (2015). Effect of the non-specific matrix metalloproteinase inhibitor Doxycycline on endometriotic implants in an experimental rat model. Exp. Ther. Med..

[22] Thorgeirsdottir T.Ó., Thormar H., Kristmundsdottir T. (2005). The influence of formulation variables on stability and microbicidal activity of monoglyceride monocaprin. J. Drug Deliv. Sci. Technol..

[23] Patlolla V.G.R., Holbrook W.P., Gizurarson S., Kristmundsdottir T. (2019). Evaluation of in vitro mucoadhesiveness and Texture Profile Analysis of doxycycline in situ hydrogels. Pharmazie.

[24] Korsmeyer R.W., Gurny R., Doelker E., Buri P., Peppas N.A. (1983). Mechanisms of solute release from porous hydrophilic polymers. Int. J. Pharm..

[25] Peter G.A. (1964). Tetracycline Formulations Stabilized by Bisulfites. U.S. Patent.

[26] Power D.F., Fieldson G., Chang Y. (2010). Tetracycline Stabilizing Formulations. U.S. Patent.

[27] Zhang H., Chen M., He Z., Wang Z., Zhang M., Wan Q., Liang D., Repka M.A., Wu C. (2013). Molecular modeling-based inclusion mechanism and stability studies of doxycycline and hydroxypropyl-beta-cyclodextrin complex for ophthalmic delivery. AAPS PharmSciTech.

[28] Nozawa S., Akazawa Y., Yasui S., Yagyu M. (1974). Aqueous Doxycycline Compositions. U.S. Patent.

[29] Skidmore R., Kovach R., Walker C., Thomas J., Bradshaw M., Leyden J., Powala C., Ashley R. (2003). Effects of Subantimicrobial-Dose Doxycycline in the Treatment of Moderate Acne. Arch. Dermatol..

[30] Bostanci N., Belibasakis G.N. (2012). Doxycycline inhibits TREM-1 induction by Porphyromonas gingivalis. FEMS Immunol. Med. Microbiol..

[31] Thomas J., Walker C., Bradshaw M. (2000). Long-term use of subantimicrobial dose doxycycline does not lead to changes in antimicrobial susceptibility. J. Periodontol..

[32] Walker C., Preshaw P.M., Novak J., Hefti A.F., Bradshaw M., Powala C. (2005). Long-term treatment with sub-antimicrobial dose doxycycline has no antibacterial effect on intestinal flora. J. Clin. Periodontol..

[33] Holbrook W.P., Kristmundsdóttir T., Thormar H., Jónsdóttir S.Á., Helgadótir H. (2014). OI0259 A denture adhesive containing monocaprin for reducing contamination with Candida. Oral. Surg. Oral Med. Oral Pathol. Oral Radiol..

[34] Giuliano E., Paolino D., Fresta M., Cosco D. (2018). Mucosal Applications of Poloxamer 407-Based Hydrogels: An Overview. Pharmaceutics.

[35] Skulason S. (2009). Bioadhesive Drug Deivery Systems in the Treatment of Oral Conditions Including Cold Sores and Aphthous Ulcers. Ph.D. Thesis.

[36] ICH Harmonised Tripartite Guideline Validation of Analytical Procedures: Text and Methodology Q2(R1). https://www.ich.org/fileadmin/Public_Web_Site/ICH_Products/Guidelines/Quality/Q2_R1/Step4/Q2_R1__Guideline.pdf.

[37] Skulason S., Asgeirsdottir M.S., Magnusson J.P., Kristmundsdottir T. (2009). Evaluation of polymeric films for buccal drug delivery. Pharmazie.

[38] Amasya G., Karavana S.Y., Sen T., Baloglu E., Tarimci N. (2012). Bioadhesive and mechanical properties of triamcinolone acetonide buccal gels. Turk. J. Pharm. Sci..

[39] Jones D.S., Woolfson A.D., Brown A.F. (1997). Textural analysis and flow rheometry of novel, bioadhesive antimicrobial oral gels. Pharm. Res..

[40] Oliveira Cardoso V.M., Stringhetti Ferreira Cury B., Evangelista R.C., Daflon Gremiao M.P. (2017). Development and characterization of cross-linked gellan gum and retrograded starch blend hydrogels for drug delivery applications. J. Mech. Behav. Biomed. Mater..

[41] Sezer A.D., Cevher E., Hatipoglu F., Ogurtan Z., Bas A.L., Akbuga J. (2008). Preparation of fucoidan-chitosan hydrogel and its application as burn healing accelerator on rabbits. Biol. Pharm. Bull..

[42] Bourne M.C. (1978). Texture Profile Analysis. Food Technol..

[43] Szczesniak A.S. (1975). General foods texture profile revisited—Ten years perspective. J. Texture Stud..

[44] Zhang L., Parsons D.L., Navarre C., Kompella U.B. (2002). Development and in-vitro evaluation of sustained release poloxamer 407 (P407) gel formulations of ceftiofur. J. Control Release.

[45] Abdelbary G.A., Aburahma M.H. (2015). Oro-dental mucoadhesive proniosomal gel formulation loaded with lornoxicam for management of dental pain. J. Liposome Res..

[46] Dash S., Murthy P.N., Nath L., Chowdhury P. (2010). Kinetic modeling on drug release from controlled drug delivery systems. Acta Pol. Pharm..

